# Extensive chronic perianal pyoderma associated with persistent inflammation caused by wearing the same unwashed underwear for more than ten years

**DOI:** 10.1002/ccr3.7477

**Published:** 2023-06-20

**Authors:** Michiko Matsuzawa Adachi, Katsuyuki Yoshida, Takahiko Fukuchi, Akira Tanaka, Naoto Yamamoto, Hitoshi Sugawara

**Affiliations:** ^1^ Department of Comprehensive Medicine 1, Division of General Medicine Jichi Medical University, Saitama Medical Centre Saitama Japan; ^2^ Department of Diagnostic Pathology Jichi Medical University, Saitama Medical Centre Saitama Japan; ^3^ Department of Comprehensive Medicine 2, Division Plastic surgery Jichi Medical University, Saitama Medical Centre Saitama Japan

**Keywords:** hidradenitis suppurativa, hygiene, molecular targeted therapy, perineum, pyoderma

## Abstract

**Key Clinical Message:**

Poor personal hygiene wearing the same unwashed briefs, and prolonged sitting have led to the development of chronic perianal pyoderma. This can be confused with hidradenitis suppurativa and must be differentiated as their treatments are different.

**Abstract:**

There are potential risks of persistent inflammation resulting from poor personal hygiene. This comprises wearing the same unwashed briefs and prolonged sitting posture that led to developing chronic perianal pyoderma (CPP) in a smoking man. CPP can be confused with hidradenitis suppurativa, requiring differentiation as their treatment strategies distinctly differ.

## 1 CLINICAL CASE

A 48‐year‐old Japanese man presented to the emergency department with worsening dyspnea. He had a four‐year history of progressive skin lesions on his gluteal region which caused bleeding and pus discharge. The patient experienced dyspnea for 2 years during winter, which hindered his ability to ride bicycle for his job due to buttock pain. He reported wearing the same unwashed briefs for over a decade, during which he did not bathe regularly but showered occasionally to wash off pus and bloody exudate. Due to the large amount of exudate, a towel was placed inside his briefs, which was changed as needed. He had a 28‐year history of smoking half a pack of cigarettes and alcohol consumption (1000 mL) q1d.

**FIGURE 1:**
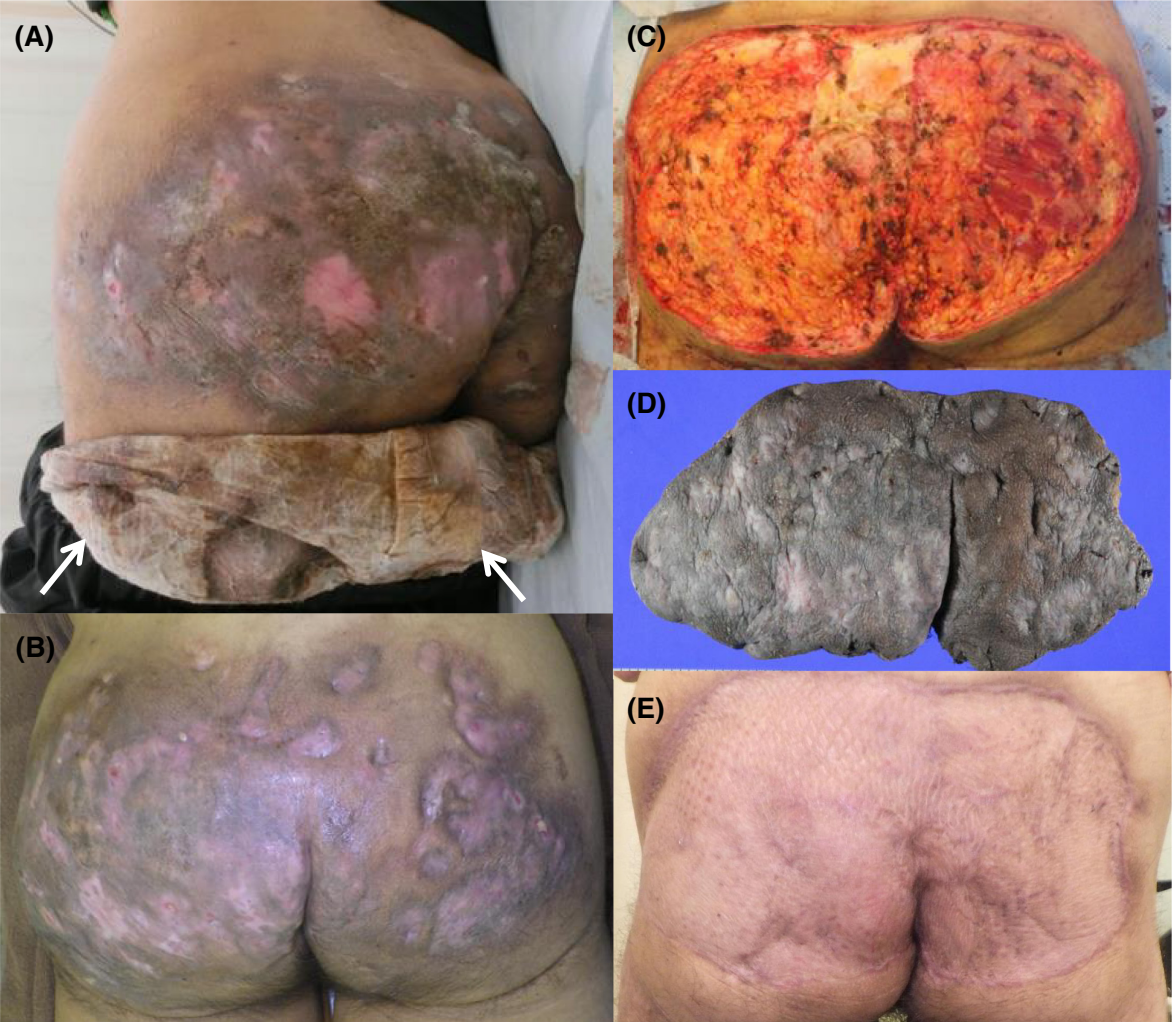
Clinical images of skin lesions on the buttocks. Panels A and B depict multiple dark, granulomatous nodules with abscesses, fistulas, and bloody pus‐like exudates that are localized to the bilateral buttocks. The white arrows in panel A indicate the briefs soiled with foul‐smelling pus upon admission. Panel C displays the affected area after wide excision, while panel D shows the resected skin lesions, resembling a seat pad. Panel E demonstrates complete healing of the skin lesions 6 months after covering with the split skin graft from the dorsal thigh.

On examination, the patient showed regular pulse, 98 beats/min; blood pressure, 169/100 mmHg; body temperature, 36.9°C; respiratory rate, 22 breaths/min; and oxygen saturation, 94% (on ambient air). The examination revealed pale conjunctiva, tooth decay, coarse crackles in the right lower lung zone, and bilateral pitting ankle edema. Skin examination showed dark, indurated, and multiple granulomatous nodules with abscesses, fistulas, and bloody pus‐like exudates localized to the bilateral buttocks (Figure 1A,B). The briefs were soiled with foul‐smelling pus (arrows in Figure 1A). No perianal skin lesions were observed. The patient was diagnosed with hypertensive heart disease, congestive heart failure, chronic kidney disease, microcytic hypochromic anemia, and extensive chronic perianal pyoderma (CPP).

**FIGURE 2:**
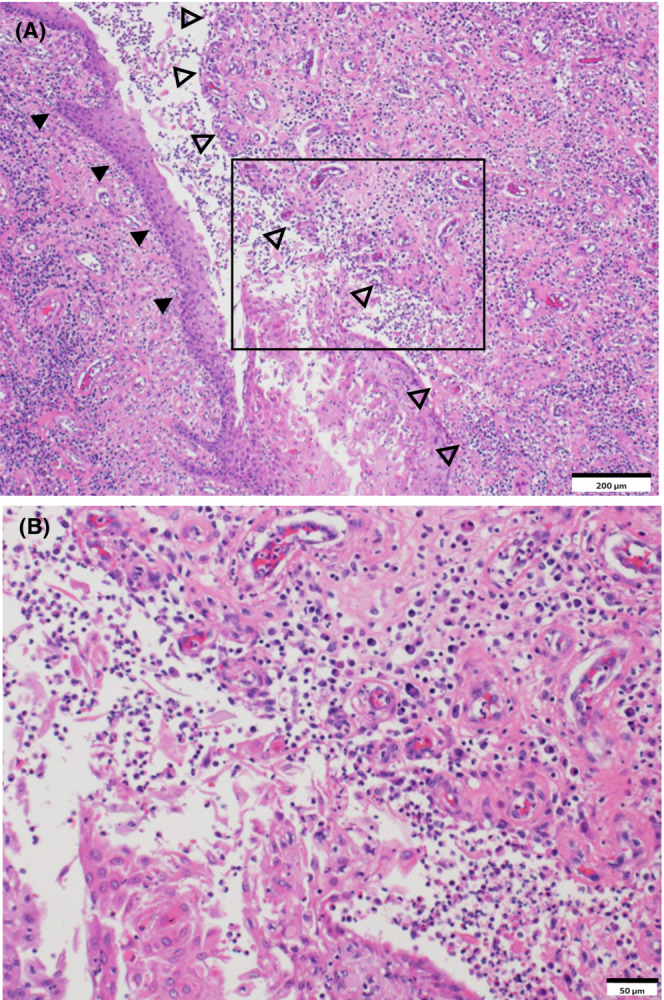
Histopathology of the skin lesion. Panel A: A low‐power photomicrograph shows the epidermis perforating into the dermis (black arrowheads) and inflammatory granulation tissue (open arrowheads) in the dermis (hematoxylin–eosin stain [H & E], bar = 200 μm). The black square indicates the high‐power view area. Panel B: A high‐power photomicrograph demonstrates the dermis exhibiting a high degree of exudative sclerosis with lymphocytic infiltration (H & E, bar = 50 μm). These findings confirm the diagnosis of chronic perianal pyoderma.

The patient was initially treated with tazobactam/piperacillin (4.5 g every 8 h for 8 days). After detecting *Proteus mirabilis* in pus, the treatment was switched to ampicillin (2 g every 6 h for 14 days). He underwent radical excision of skin and subcutaneous tissues of the gluteal superficial fascia (Figure 1C). Moreover, he received vacuum‐assisted closure for 6 days. The resected specimen resembled a seat pad (Figure 1D). Histopathological findings confirmed the diagnosis of CCP (Figure 2). 2 weeks later, a split skin grafting from the left dorsal thigh meshed in a 1.5:1 ratio was applied to the well‐granulating lesion. Complete healing was achieved after 6 months (Figure 1E).

This case highlights the potential risks associated with poor personal hygiene and prolonged sitting posture, which could lead to CPP. CPP is a rare condition that was first reported in 1969 by Möller^1^ and could typically affect the dorsal parts of the buttocks in men.^2^ It may lead to developing squamous cell carcinoma.^2^ Several case reports have described the need for extensive excision and skin implantation.^2^ The prevalence of CPP remains not well known due to confusion with or inclusion in hidradenitis suppurativa (HS).^2^ HS is a chronic and recurrent inflammatory disease affecting the follicular portion of folliculopilosebaceous units, typically the intertriginous sites including the axillary, breast, and anogenital apocrine glands. It often coexists with inflammatory bowel disease and is estimated to affect 1% of the population and more common in women with obesity.^3^ Potential treatments of HS include various molecular targeted therapies and adalimumab.^3^ Physicians should carefully distinguish between CPP and HS, as they require different treatments.

## AUTHOR CONTRIBUTIONS

**Michiko Matsuzawa Adachi:** Conceptualization; data curation; investigation; writing – original draft. **Katsuyuki Yoshida:** Investigation; supervision. **Takahiko Fukuchi:** Supervision; writing – review and editing. **Akira Tanaka:** Investigation; supervision; writing – review and editing. **Naoto Yamamoto:** Investigation; supervision; writing – review and editing. **Hitoshi Sugawara:** Conceptualization; investigation; project administration; supervision; visualization; writing – original draft; writing – review and editing.

## FUNDING INFORMATION

No funding was received for this study.

## CONFLICT OF INTEREST STATEMENT

The authors declare no competing interests.

## CONSENT

Written informed consent was obtained from the patient.

## Data Availability

The datasets generated and analyzed will be available upon request to the corresponding author.
